# Patterns and clinical outcomes of injuries related to two-wheeled vehicles (bicycle and motorcycle) in the geriatric population: a nationwide analysis in South Korea (2016–2018)

**DOI:** 10.1186/s12877-021-02505-2

**Published:** 2021-10-26

**Authors:** Yoonhyung Choi, Duk Hee Lee, Jung Il Lee

**Affiliations:** 1 Department of Emergency Medicine, College of Medicine, Ewha Womans University, Seoul, South Korea; 2 Department of Orthopedic Surgery, Korea University Guro Hospital, Seoul, South Korea

**Keywords:** Bicycle, Motorcycle, Two-wheeled vehicle, Injury pattern, Injury severity score, Geriatric trauma

## Abstract

**Background:**

South Korea has a rapidly ageing population. This study aimed to provide epidemiologic data and to identify the characteristics of the patterns and clinical outcomes of two-wheeled vehicle-related injuries (bicycle and motorcycle) in elderly riders.

**Methods:**

This study retrospectively analyzed data from the National Emergency Department Information System from 2016 to 2018. Adult patients (≥ 20 years old) who were injured while using two-wheeled vehicles were included. Elderly patients were defined as being 65 years and older. The analysis was performed for 65,648 bicycle-related injuries (15,272 elderly patients) and 87,855 motorcycles-related injuries (17,292 elderly patients).

**Results:**

In emergency departments (EDs), the average injury severity score (ISS) for motorcycle-related accidents was 9.8 ± 11.2 in the younger group and 14.1 ± 14.7 in the elderly group (*p* = 0.001). In addition, the average ISS of bicycle-related accidents was 7.1 ± 8.9 in the younger group and 10.5 ± 12.3 in the elderly group (*p* = 0.001). Two-wheeled vehicle accident mortality rates of elderly riders (0.9% for bicycle and 1.8% for motorcycle in the ED; 4.1% for bicycle and 3.8% for motorcycle in the hospital) were more than twice those of younger riders.

The elderly stayed in the hospital longer than younger patients (485.2 ± 543.0 h vs 336.8 ± 385.5 h, *p* = 0.001) for bicycle-related injuries. They also stayed longer for motorcycle-related injuries (529 ± 598.6 h vs 452.0 ± 543.55 h, *p* = 0.001).

The logistic regression analysis showed that age ≥ 65 years was an independent factor associated with severe trauma (ISS ≥ 16) for both bicycle-related injuries (adjusted odds ratio [OR] 2.185 [95% Confidence Interval (CI) 2.072–2.303]) and motorcycle-related injuries (adjusted OR 1.220 [95% CI 1.137–1.287]).

**Conclusion:**

Two-wheeled vehicle-related injuries in the elderly were associated with higher ISS, length of hospital stay, and mortality than in younger riders. Analysing the characteristics of two-wheeled vehicle-related injuries in the elderly can be the basis for planning to reduce and prevent injuries in elderly riders.

## Background

Bicycles and motorcycles are important and affordable transportation devices. The use of bicycles for urban transportation and leisure activity and of motorcycles for transportation and on-demand delivery is increasing. Approximately 800 million people worldwide use bicycles as a means of transportation [1–3]. Bicycles offer many advantages by promoting an interest in health and participation in leisure activities, lowering fuel-related air pollution, and reducing traffic caused by automobiles. Therefore, countries such as the Netherlands, Denmark, the United States, and Germany have implemented policies to encourage bicycle use [4–6]. In South Korea, the Act on Activation of Bicycle Use, enacted in 1995, has been amended more than 10 times, and by enacting enforcement decrees and regulations, efforts to further encourage bicycle use and reduce the risk of accidents are ongoing. However, the definition of bicycle roads is still unclear in South Korea, compared with the aforementioned countries, resulting in a combination of bicycle-pedestrian roads and bicycle-car roads. There are no rules pertaining to bicycle safety equipment (such as bicycle bells) and no traffic signs for bicycles. Bike crossings must also be made more secure [7, 8]. The rate of bicycle use is increasing owing to the government policy on the promotion and convenience of bicycle use [9, 10]. According to the National Statistical Office, each household in South Korea owns 1.6 bicycles, and one-third of the population uses bicycles [11]. Meanwhile, as the usage rate increses, the number of bicycle accidents does as well. According to Korea Road Traffic Authority data, there were 12,000–17,000 bicycle accidents per year from 2011 to 2015. Although there was a slight decrease in 2016 and 2017, the number of deaths was approximately 250 per year from 2011 to 2017. The data also show that the proportion of bicycle accident deaths gradually increased over the course of 7 years, from 5.3 to 6.3% [12].

The fatality rates of motorcycle accidents were higher than those of other means of transportation due to the limited safety precautions and the difference in the mechanism of injury [13]. Studies have shown that age, helmet use, alcohol consumption, speed, road surface condition, road safety barriers, engine displacement, collision objects, and weather affect the severity of motorcycle accidents [14–17]. The severity and mortality rate of traffic accidents vary significantly from country to country because they are affected by the unique topography and transportation system of each country and the vehicle type used. As of 2015, South Korea ranked second highest in traffic accidents among member countries of the Organisation for Economic Co-operation and Development with 455 traffic accidents per 100,000 population and fourth in traffic accident deaths with 9.1 per 100,000 population [18]. Road traffic-related crashes are the leading cause of injuries requiring hospitalization, and bicyclists and motorcyclists contribute to the morbidity and mortality rates of road crash casualties [19]. The fatality rates of traffic accidents while riding a motorcycle or walking are higher in South Korea than in other countries [20].

In 2018, 14.4% of the total population of South Korea was ≥65 years old. This proportion is expected to increse to 20.3% in 2025 and 46.5% in 2067 [21]. Moreover, there is increasing number of elderly cyclists and motorcyclists, and this trend is expected to continue. Elderly riders may have more risk factors and problems associated with cycling as they may have decreased peripheral vision, coordination, balance, and cognitive function [22]. However, there have been no studies using data from emergency departments (EDs) and hospitals in a nationwide database to compare the different characteristics of two-wheeled vehicle-related injuries in older and younger riders.

Understanding such differences, including their incidence and type, could contribute significantly to establishing safety policies. Hence, this study aimed to provide epidemiologic data and to identify characteristics of the patterns and clinical outcomes of two-wheeled vehicle-related (bicycle and motorcycle) injuries in elderly riders.

## Methods

### Setting and data collection

This retrospective study used the data collected from the National Emergency Department Information System (NEDIS) from January 2016 to December 2018. NEDIS transmits data to the National Emergency Medical Centre’s server operated under the supervision of the Korean Ministry of Health and Welfare. Patient information is transferred automatically in real time from all South Korean EDs nationwide. To revitalize the use of emergency medical information, the Korean Ministry of Health and Welfare established the NEDIS database in 2001. This information is qualified as accurate, and the results are reported by the Ministry of Health and Welfare on an annual basis.

There are 36 regional emergency medical centres (Level 1), 117 local emergency medical centres (Level 2), and 119 local emergency medical rooms (Level 3) in South Korea. Between 2016 and 2018, 399 out of 401 emergency medical institutions participated in NEDIS data collection [23].

### Variables and outcome measures

NEDIS collects demographic and clinical data: age, sex, ED visit date, ED visit time, geographic location of ED, insurance type, helmet use, means of visit, consciousness of patients in the ED (alert mental state and altered mental state, including verbal responses, pain responses, and unresponsiveness), systolic blood pressure, diastolic blood pressure, pulse rate, respiratory rate, diagnosis in the ED, injury severity score (ISS), and disposition after ED care (discharge, transfer to another hospital, admission to general ward [GW] or intensive care unit [ICU]). For admitted patients, the final diagnosis data and medical results on discharge were considered for the study. We divided ED visit dates by season: spring (March to May), summer (June to August), autumn (September to November), and winter (December to February). The time of the visit was divided into dawn (00:00 to 05:59 h), morning (06:00 to 11:59 h), afternoon (12:00 to 17:59 h), and night (18:00 to 23:59 h).

Injured patients aged ≥20 years old were included, and we defined the elderly as patients aged ≥65 years. Urban areas included eight cities and their metropolitan areas, and rural areas included seven provinces and Jeju Island.

During the study period, there were 212,112 two-wheeled vehicle-related injuries. In South Korea, drivers’ licenses are issued to adults aged ≥20 years; therefore, patients under 19 were excluded. A total of 4267 patients with missing or incomplete data were excluded.

**Fig. 1:**
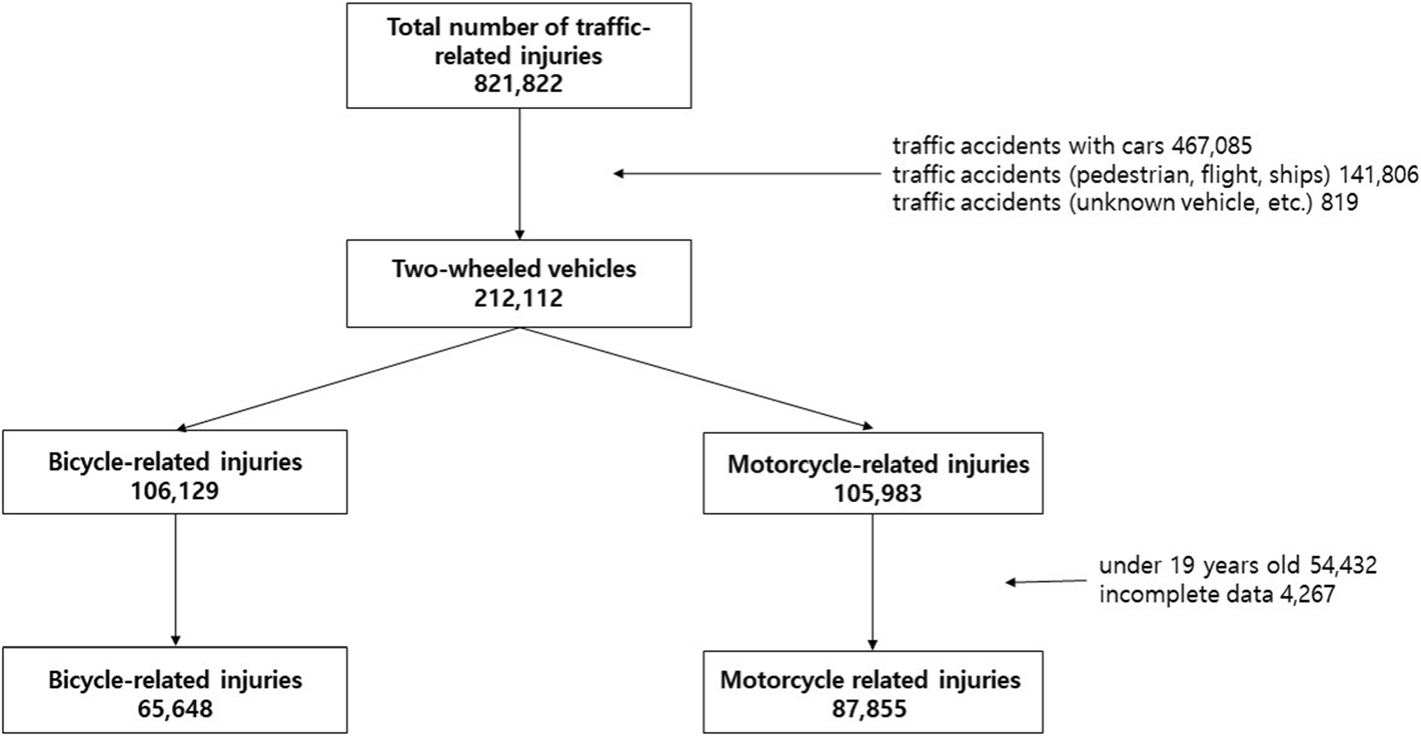
The study flow diagram of the enrolled patients

The younger group under 65 years of age and the elderly group aged 65 and older were compared. The final analysis was performed on 65,648 bicycles-related injuries (15,272 elderly patients) and 87,855 motorcycles-related injuries (17,292 elderly patients). Figure 1 shows the study flow chart for enrolled patients. We analysed the final diagnosis to categorise the injury (injury regions and fracture sites) according to the International Classification of Diseases, Tenth Revision [24]. ED disposition, duration of hospitalisation, and mortality were analysed to determine the clinical outcomes in both younger and elderly riders.

### Statistical analysis

We analysed and compared the variables of the patients and injury-related characteristics between the younger and elderly groups. Categorical variables were analysed with the chi-square test, and Student’s *t*-test was used for continuous variables. Gender, season of injury, time of injury, helmet use, age group (younger and elderly), and area (urban or rural) showed significant differences between ISS ≤ 15 and ISS ≥ 16 in the univariate analysis. To investigate the factors predicting severe trauma (ISS ≥ 16), a multivariable logistic regression analysis was performed using these factors for each bicycle and motorcycle group. A two-tailed *p*-value of < 0.05 was considered statistically significant. The Statistical Package for the Social Sciences Statistics for Windows, version 21 (International Business Machines Corporation, Armonk, NY, USA) was used for the analysis.

## Results

### Incidence of Korean traffic accidents and two-wheeled vehicle-related (bicycle and motorcycle) related injuries (Table 1)

The annual total incidence of patients with traffic injuries admitted to EDs showed an increasing trend from 2016 to 2018. While bicycle-related injuries decreased among younger riders, motorcycle-related injuries increased. The number of elderly riders with two-wheeled vehicle-related injuries increased.

**Table 1:** Incidence of Korean traffic accidents and two-wheeled vehicle-related (bicycle and motorcycle) related injuries in the emergency department (2016–2018)

		2016		2017		2018		Total	
**Korean population by age**	Younger	34,653,358		34,682,815		34,741,436		104,077,609	
	Elderly	6,995,652		7,356,106		7,650,408		22,002,166	
		incidence	Number of injuries/100,000 population	incidence	number of injuries/100,000 population	incidence	number of injuries/100,000 population	incidence	number of injuries/100,000 population
**Total traffic injuries**	Younger	186,803	539.1	192,764	555.8	189,479	545.4	569,046	546.8
	Elderly	35,462	506.9	40,524	550.9	40,924	534.9	116,910	531.4
**Bicycle-related injuries**	Younger	17,362	50.1	17,347	50.0	15,667	45.1	50,376	48.4
	Elderly	4664	66.7	5347	72.7	5262	68.8	15,272	69.4
**Motorcycle-related injuries**	Younger	22,996	66.4	23,649	68.2	23,918	68.8	70,563	67.8
	Elderly	5454	78.0	6053	82.3	5785	75.6	17,292	78.6

Elderly, ≥ 65 years old

Younger, 20 ~ 64 years old

### Demographic characteristics of patients who visited EDs with two-wheeled vehicle related injuries (Table 2)

There were 65,648 bicycle-related injuries (50,376 younger and 15,272 elderly riders). There were 35,906 (71.3%) males among the younger riders and 12,406 (81.2%) males among the elderly riders (*p* < 0.001). The analysis of the means of visit showed that younger bicyclists presented themselves to the ED, whereas elderly riders were usually transported by 911 emergency services. Younger bicycle riders wore helmets (29.9%) more than elderly riders (10.5%)(*p* < 0.001). The incidence of bicycle accidents per 100,000 population was 69.4 in the elderly group (72.3 in urban, 64.6 in rural). This was higher than the incidence of 48.4 in the younger group which had more frequent accidents in urban areas (*p* < 0.001). There were more patients with altered consciousness in the elderly group than in the younger group (*p* < 0.001).

**Table 2:** Demographic characteristics of patients who visited the emergency department with two-wheeled vehicle-related injuries

Variable	Bicycle				Motorcycle			
	Younger	Elderly	Total	***p-value***	Younger	Elderly	Total	***p-value***
**Number of injury cases**	50,376	15,272	65,648		70,563	17,292	87,855	
**Age**	43.3 ± 13.3	73.7 ± 6.1	50.4 ± 17.6	0.0001	37.4 ± 13.1	74.3 ± 5.9	44.7 ± 19.0	0.001
**Number of males (%)**	35,906 (71.3%)	12,406 (81.2%)	48,312 (73.6%)	0.0001	64,112 (90.9%)	14,196 (82.1%)	78,308 (89.1%)	0.001
**Season of injury**				0.0001				0.001
Spring	13,702 (27.2%)	3838 (25.1%)	17,540 (26.7%)		16,846 (23.9%)	4505 (26.1%)	21,351 (24.3%)	
Summer	17,031 (33.8%)	4564 (29.9%)	21,595 (32.9%)		20,024 (28.4%)	5037 (29.1%)	25,061 (28.5%)	
Autumn	14,690 (29.2%)	4582 (30.0%)	19,272 (29.4%)		19,796 (28.1%)	4902 (28.3%)	24,698 (28.1%)	
Winter	4.953 (9.8%)	2288 (15.0%)	7241 (11.0%)		13,897 (19.7%)	2848 (16.5%)	16,745 (19.1%)	
**Time of injury**				0.0001				0.001
Morning	10,698 (21.2%)	4711 (30.8%)	15,409 (23.5%)		10,441 (14.8%)	4945 (28.6%)	15,386 (17.5%)	
Afternoon	15,982 (31.7%)	6427 (42.1%)	22,409 (34.1%)		22,068 (31.3%)	7941 (45.9%)	30,009 (34.2%)	
Night	19,111 (37.9%)	3677 (24.1%)	22,788 (34.7%)		29,356 (41.6%)	3995 (23.1%)	33,351 (38.0%)	
Dawn	4585 (9.1%)	457 (3.0%)	5042 (7.7%)		8698 (12.3%)	411 (2.4%)	9109 (10.4%)	
**Helmet use**				0.0001				0.001
Yes	15,062 (29.9%)	1604 (10.5%)	16,666 (25.4%)		56,944 (80.7%)	10,790 (62.4%)	67,734 (77.1%)	
No	35,314 (70.1%)	13,668 (89.5%)	48,982 (74.6%)		13,619 (19.3%)	6502 (37.6%)	20,121 (22.9%)	
**Insurance type**				0.0001				0.001
National health care	33,295 (66.1%)	7028 (46.0%)	40,323 (61.4%)		15,972 (22.6%)	5671 (32.8%)	21,643 (24.6%)	
Traffic accident insurance	14,623 (29.0%)	7415 (48.6%)	22,038 (33.6%)		50,164 (71.1%)	10,434 (60.3%)	60,598 (69.0)	
Industrial accident insurance	34 (0.1%)	6 (0.0%)	40 (0.1%)		319 (0.5%)	17 (0.1%)	336 (0.4%)	
Medicaid	1320 (2.6%)	587 (3.8%)	1907 (2.9%)		1220 (1.7%)	564 (3.3%)	1784 (2.0%)	
Others	1104 (2.2%)	236 (1.5%)	1340 (2.0%)		2888 (4.1%)	606 (3.5%)	3494 (4.0%)	
**Means of visit**				0.0001				0.001
911	20,638 (41.0%)	8448 (55.3%)	29,086 (44.3%)		43,109 (61.1%)	10,374 (60.0%)	53,483 (60.9%)	
Hospital ambulance	1676 (3.3%)	1268 (8.3%)	2944 (4.5%)		3447 (4.9%)	2842 (16.4%)	6289 (7.2%)	
Patient visit	27,920 (55.4%)	5486 (35.9%)	33,406 (50.9%)		23,733 (33.6%)	3911 (22.6%)	27,644 (31.5%)	
Others	142 (0.3%)	70 (0.5%)	212 (0.3%)		274 (0.4%)	165 (1.0%)	439 (0.5%)	
**Area**				0.0001				0.001
Urban area	37,694 (74.8%)	9928 (65.0%)	47,622 (72.5%)		51,316 (72.7%)	7524 (43.5%)	58,840 (67.0%)	
*Number of urban injuries /100,000 population*	50.9	72.3	54.3		69.3	54.8	67.0	
Rural area	12,682 (25.2%)	5344 (35.0%)	18,026 (27.5%)		19,247 (27.3%)	9768 (56.5%)	29,015 (33.0%)	
*Number of rural injuries /100,000 population*	42.2	64.6	47.1		64.1	118.1	75.7	
**Consciousness**				0.0001				0.001
Alert	49,487 (98.2%)	14,417 (94.4%)	63,904 (97.4%)		67,615 (95.8%)	15,649 (90.5%)	83,264 (94.8%)	
Altered	884 (1.8%)	852 (5.6%)	1736 (2.6%)		2944 (4.2%)	1641 (9.5%)	4585 (5.2%)	
**Systolic blood pressure (mmHg)**	133.4 ± 26.8	142.6 ± 31.6	135.6 ± 28.2	0.0001	134.4 ± 29.6	140.4 ± 34.8	135.6 ± 30.8	0.001
**Diastolic blood pressure (mmHg)**	81.4 ± 21.3	81.76 ± 20.9	81.4 ± 21.2	0.109	81.6 ± 21.4	80.8 ± 23.0	81.4 ± 21.7	0.001
**Pulse rate (beats/min)**	82.0 ± 21.0	79.7 ± 21.0	81.4 ± 20.8	0.0001	83.4 ± 20.6	80.2 ± 22.2	82.8 ± 21.0	0.001
**Respirate rate (/min)**	19.37 ± 6.6	19.3 ± 4.1	19.4 ± 6.0	0.606	19.3 ± 13.7	19.3 ± 15.4	19.3 ± 4.1	0.622
**Body temperature (°C)**	36.5 ± 2.6	36.6 ± 3.5	36.4 ± 2.8	0.0001	36.2 ± 4.1	36.1 ± 4.3	36.2 ± 4.2	0.009

Elderly, ≥ 65 years old

Younger, 20 ~ 64 years old

There were 87,855 motorcycle-related injuries (70,563 younger and 17,292 elderly riders). Male represented the majority among both younger (90.9%) and elderly (81.2%) riders (*p* = 0.001). Helmet use was more prevalent among younger riders (80.7%) than among elderly riders (62.4%) (*p* < 0.001). The incidence of motorcycle accidents per 100,000 population was 78.6 in the elderly group (54.8 urban, 118.1 rural), which was higher than the incidence in the younger group (67.8) (*p* < 0.001). Altered consciousness was more common in the elderly riders (9.5%) than in younger riders (4.2%) (*p* = 0.001).

### Injury patterns and clinical outcomes of patients who visited EDs with two-wheeled vehicle-related injuries (Table 3)

Bicycle-related injuries mainly involved the external body surface. The second most frequently injured area being the upper extremities in younger riders and the head and neck in elderly riders. Younger riders tended to break their forearm and clavicle, whereas elderly riders broke their forearm and femur. The average ISS was 7.1 ± 8.9 for younger riders and 10.5 ± 12.3 for elderly riders (*p* = 0.001). The percentage of ISS ≥ 16 points was higher in the elderly group than in the younger group (22.7% vs 12.5%, *p* = 0.001). The majority of younger riders (77.6%) were discharged from the ED, 16.8% were admitted to the GW, and 2.4% were admitted to the ICU. In contrast, 57.2% of elderly riders were discharged from the ED, whereas 29.3% were admitted to the GW and 7.7% to the ICU. The mortality rate of elderly riders was higher than that of younger riders (0.9% vs 0.2%).

**Table 3:** Injury patterns and clinical outcomes of patients who visited the emergency department with two-wheeled vehicle-related injuries

Variable	Bicycle							Motorcycle						
	Younger		Elderly		Total		***p-value***	Younger		Elderly		Total		***p-value***
**Number of patients**	50,376		15,272		65,648			70,563		17,292		87,855		
**Injury pattern**														
**Region of injury**														
Head and neck	11,178	22.2%	5927	38.8%	17,105	26.1%		20,099	28.5%	8931	51.6%	29,030	33.0%	
Face	5153	10.2%	1397	9.1%	6550	10.0%		6062	8.6%	2364	13.7%	8426	9.6%	
Chest	5786	11.5%	2782	18.2%	8568	13.1%		11,607	16.4%	5044	29.2%	16,651	19.0%	
Abdomen and pelvic contents	438	0.9%	178	1.2%	616	0.9%		1559	2.2%	479	2.8%	2038	2.3%	
External	35,174	69.8%	9592	62.8%	44,766	68.2%		50,788	72.0%	11,110	64.2%	61,898	70.5%	
pelvic girdle	361	0.7%	302	2.0%	663	1.0%		968	1.4%	386	2.2%	1354	1.5%	
Upper extremities	17,342	34.4%	3316	21.7%	20,658	31.5%		22,452	31.8%	4832	27.9%	27,284	31.1%	
Lower extremities	11,471	22.8%	3962	25.9%	15,433	23.5%		32,737	46.4%	5673	32.8%	38,410	43.7%	
Spine	2546	5.1%	1345	8.8%	3891	5.9%		5185	7.3%	1209	7.0%	6394	7.3%	
**Number of fractures**	28,025		9331		37,356			45,524		14,894		60,418		
**Fracture site**														
Skull	950	3.4%	504	5.4%	1454	3.9%		1910	4.2%	955	6.4%	2865	4.7%	
Face	4405	15.7%	1275	13.7%	5680	15.2%		5518	12.1%	2210	14.8%	7728	12.8%	
Spine	3366	12.0%	1556	16.7%	4922	13.2%		6665	14.6%	1784	12.0%	8449	14.0%	
Rib, sternum	2369	8.5%	1334	14.3%	3703	9.9%		4840	10.6%	2834	19.0%	7674	12.7%	
Scapula	1433	5.1%	369	4.0%	1802	4.8%		1831	4.0%	690	4.6%	2521	4.2%	
Clavicle	2725	9.7%	357	3.8%	3082	8.3%		2616	5.7%	776	5.2%	3392	5.6%	
Humerus	797	2.8%	220	2.4%	1017	2.7%		884	1.9%	293	2.0%	1177	1.9%	
Forearm	7605	27.1%	1406	15.1%	9011	24.1%		9691	21.3%	1781	12.0%	11,472	19.0%	
Hand	1724	6.2%	293	3.1%	2017	5.4%		2260	5.0%	527	3.5%	2787	4.6%	
Pelvic ring	256	0.9%	233	2.5%	489	1.3%		598	1.3%	274	1.8%	872	1.4%	
Acetabulum	72	0.3%	58	0.6%	130	0.3%		266	0.6%	99	0.7%	365	0.6%	
Femur	817	2.9%	1034	11.1%	1851	5.0%		1764	3.9%	768	5.2%	2532	4.2%	
Patellar	3	0.0%	1	0.0%	4	0.0%		15	0.0%	2	0.0%	17	0.0%	
Tibia/fibula	1012	3.6%	547	5.9%	1559	4.2%		4437	9.7%	1490	10.0%	5927	9.8%	
Foot	491	1.8%	144	1.5%	635	1.7%		2229	4.9%	411	2.8%	2640	4.4%	
**ISS**	7.1 ± 8.9		10.5 ± 12.3		7.9 ± 9.9		0.001	9.8 ± 11.2		14.1 ± 14.7		10.6 ± 12.1		0.001
**ISS ≥ 16**	6297	12.5%	3467	22.7%	9764	14.8%	0.001	14,747	20.9%	5741	33.2%	20,488	23.2%	0.001
**ED disposition**														
Discharge	39,093	77.6%	8734	57.2%	47,827	72.9%		48,527	68.8%	6850	39.6%	55,377	63.0%	
Transfer	1365	2.7%	715	4.7%	2080	3.2%		2874	4.1%	1326	7.7%	4200	4.8%	
ICU admission	1234	2.4%	1177	7.7%	2411	3.7%		3675	5.2%	2480	14.3%	6155	7.0%	
GW admission	8449	16.8%	4471	29.3%	12,920	19.7%		14,738	20.9%	6301	36.4%	21,039	23.9%	
Death	82	0.2%	143	0.9%	225	0.3%		546	0.8%	304	1.8%	850	1.0%	
Others	129	0.3%	26	0.2%	155	0.2%		178	0.3%	22	0.1%	200	0.2%	

Elderly, ≥ 65 years old

Younger, 20 ~ 64 years old

*ED* emergency department, *GW* general ward, *ICU* intensive care unit, *ISS* Injury severity score, *LOS*, Length of stay

Among patients with motorcycle-related injuries, younger riders mainly injured their external body surface and lower extremities, whereas elderly riders mainly injured their external body surface and head and neck areas. Younger riders mainly broke their forearm and spine, whereas elderly riders mainly broke their ribs or sternum and facial bones. The average ISS of the younger group was 9.8 ± 11.2 and that of the elderly group was 14.1 ± 14.7 (*p* = 0.001). The percentage of ISS ≥ 16 points was higher in the elderly group than in the younger group (33.2% vs 20.9%) (*p* = 0.001). Among younger riders, 68.8% were discharged, 20.9% were admitted to the GW, 4.1% were admitted to the ICU, and 0.8% died. Fewer elderly riders (39.6%) were discharged, whereas 36.4% were admitted to the GW, 14.3% were admitted to the ICU, and 1.8% died.

### Injury patterns and clinical outcomes of patients who were hospitalised with two-wheeled vehicle-related injuries (Table 4)

Among patients hospitalised for bicycle-related injuries, younger riders tended to injure their head and neck, external body surface, and upper extremities. Elderly riders mainly injured their head and neck, followed by their external body surface and lower extremities. A comparison of the fracture sites revealed that both groups suffered mainly spine injuries, followed by facial bone and rib and sternum fractures for the younger group, and rib and sternum and femur fractures for the elderly group. The average ISS was higher for elderly riders than for younger ones (19.2 ± 16.4 vs 16.3 ± 15.0) (*p* = 0.001), and more elderly riders (17%) had more severe injuries (a higher percentage of ISS ≥ 16 points) than younger riders (7.4%) (*p* = 0.001). The length of stay (LOS) in hospital was 485.2 ± 543.0 h for elderly riders, which was longer than that of younger riders (336.8 ± 385.5 h). The in-hospital mortality of elderly riders was more than three times that of younger riders (4.1% vs 1.2%) (*p* = 0.001).

**Table 4:** Injury patterns and clinical outcomes of patients who were hospitalized with two-wheeled vehicle-related injuries

Variable	Variable							Variable						
	Younger		Elderly		Total		***p-value***	Younger		Elderly		Total		***p-value***
**Number of patients**	**Number of patients**	**Number of patients**	**Number of patients**	**Number of patients**	**Number of patients**	**Number of patients**	**Number of patients**	**Number of patients**	**Number of patients**	**Number of patients**	**Number of patients**	**Number of patients**	**Number of patients**	**Number of patients**
**In-admission injury pattern**														
**Region of injury**														
Head and neck	4725	48.8%	3692	65.4%	8417	54.9%		9855	53.5%	6440	73.3%	16,295	59.9%	
Face	1660	17.1%	625	11.1%	2285	14.9%		3685	20.0%	1458	16.6%	5143	18.9%	
Chest	2735	28.2%	1728	30.6%	4463	29.1%		6605	35.9%	3955	45.0%	10,560	38.8%	
Abdomen and pelvic contents	360	3.7%	147	2.6%	507	3.3%		1530	8.3%	483	5.5%	2013	7.4%	
External	4344	44.9%	2233	39.5%	6577	42.9%		9447	51.3%	3945	44.9%	13,392	49.2%	
pelvic girdle	314	3.2%	255	4.5%	569	3.7%		860	4.7%	386	4.4%	1246	4.6%	
Upper extremities	4247	43.9%	1391	24.6%	5638	36.8%		7937	43.1%	3082	35.1%	11,019	40.5%	
Lower extremities	2964	30.6%	2044	36.2%	5008	32.7%		11,914	64.7%	3783	43.1%	15,697	57.7%	
Spine	1394	14.4%	913	16.2%	2307	15.0%		2821	15.3%	1082	12.3%	3903	14.4%	
**Number of fractures in hospitalization patients**	10,973		5796		16,769			25,751		11,138		36,889		
**Fracture site**														
Skull	637	5.8%	371	6.4%	1008	6.0%		1394	5.4%	678	6.1%	2072	5.6%	
Face	1531	14.0%	574	9.9%	2105	12.6%		3450	13.4%	1349	12.1%	4799	13.0%	
Spine	1750	15.9%	1141	19.7%	2891	17.2%		3652	14.2%	1664	14.9%	5316	14.4%	
Rib, sternum	1402	12.8%	900	15.5%	2302	13.7%		3323	12.9%	2252	20.2%	5575	15.1%	
Scapula	652	5.9%	213	3.7%	865	5.2%		1178	4.6%	516	4.6%	1694	4.6%	
Clavicle	1093	10.0%	237	4.1%	1330	7.9%		1553	6.0%	598	5.4%	2151	5.8%	
Humerus	329	3.0%	137	2.4%	466	2.8%		501	1.9%	201	1.8%	702	1.9%	
Forearm	1306	11.9%	428	7.4%	1734	10.3%		2386	9.3%	924	8.3%	3310	9.0%	
Hand	424	3.9%	166	2.9%	590	3.5%		1287	5.0%	400	3.6%	1687	4.6%	
Pelvic ring	206	1.9%	163	2.8%	369	2.2%		469	1.8%	257	2.3%	726	2.0%	
Acetabulum	89	0.8%	81	1.4%	170	1.0%		344	1.3%	113	1.0%	457	1.2%	
Femur	586	5.3%	817	14.1%	1403	8.4%		1391	5.4%	622	5.6%	2013	5.5%	
Patellar	–	0.0%	–	0.0%	–	0.0%		7	0.0%	–	0.0%	7	0.0%	
Tibia/fibula	693	6.3%	446	7.7%	1139	6.8%		3168	12.3%	1173	10.5%	4341	11.8%	
Foot	275	2.5%	122	2.1%	397	2.4%		1648	6.4%	391	3.5%	2039	5.5%	
**ISS**	16.3 ± 15.0		19.2 ± 16.4		17.3 ± 15.6		0.001	19.1 ± 16.4		21.6 ± 17.8		19.9 ± 16.9		0.001
**ISS ≥ 16**	717	7.4%	980	17.0%	1697	9.6%	0.001	2301	12.5%	2353	26.8%	4654	15.2%	0.001
**Clinical outcomes**														
**Hospital LOS (hours)**	336.8 ± 385.5		485.2 ± 543.0		391.4 ± 455.6		0.001	452.0 ± 543.55		529.6 ± 598.6		477.1 ± 563.1		0.001
**Hospital death**	121	1.2%	238	4.1%	359	2.1%	0.001	317	1.7%	418	3.8%	735	2.7%	0.001

Elderly, ≥ 65 years old

Younger, 20 ~ 64 years old

*ISS* Injury severity score, *LOS* Length of stay

Among admitted patients, motorcyclists suffered more injuries than cyclists. Younger motorcyclists injured their lower extremities and head and neck, whereas elderly motorcyclists mainly injured their head and neck and chest. Younger patients were prone to fracturing their spine and facial bones, whereas elderly patients tended to fracture their ribs and sternum as well as their spine. The average ISS was 19.1 ± 16.4 for younger riders and 21.6 ± 17.8 for elderly riders (*p* = 0.001). The percentage of ISS ≥ 16 points was 26.8% for the elderly group and 12.5% for the younger group (*p* = 0.001). Admitted motorcyclists stayed longer in the hospital than admitted bicyclists. The LOS in hospital was 452.0 ± 543.6 h for younger motorcyclists and 529.6 ± 598.6 h the elderly motorcyclists (*p* = 0.001). The in-hospital mortality rate was 1.7% the younger motorcyclists and 3.8% for the elderly motorcyclists (*p* = 0.001).

### Factors predicting severe trauma (ISS ≥ 16) in bicycle- and motorcycle-related injuries (Table 5)

The multivariable logistic regression analysis showed that the elderly group and rural areas were independent factors associated with severe trauma (ISS ≥ 16). The elderly group had an adjusted odds ratio (OR) of 2.185 (95% CI 2.072–2.303) for bicycle-related injuries and an adjusted OR of 1.220 (95% CI 1.137–1.287) for motorcycle-related injuries compared to the younger group.

**Table 5:** Factors predicting severe trauma (ISS ≥ 16) in bicycle- and motorcycle-related injuries

Outcome: ISS ≥ 16	Bicycle-related injury			Motorcycle-related injury		
	Adjusted OR	95% CI	*p-value*	Adjusted OR	95% CI	*p-value*
Sex						
Male	1.007	0.949–1.067	0.826	0.944	0.889–1.002	0..057
Age						
Elderly	2.185	2.072–2.303	**0.000**	1.220	1.137–1.287	**0.003**
Area						
Rural	1.078	1.021–1.140	**0.007**	0.999	0.953–1.046	0.959
Helmet use						
No use	1.000	0.941–1.064	0.993	0.994	0.950–1.040	0.794
Season						
Spring	1.000	Reference	0.730	1.000	Reference	0.046
Summer	0.965	0.904–1.031	0.291	1.007	0.956–1.060	0.798
Autumn	0.977	0.914–1.044	0.489	1.058	1.006–1.114	**0.029**
Winter	0.995	0.911–1.087	0.920	1.057	1.000–1.119	0.051
Time						
Morning (06:00 ~ 11:59)	1.000	Reference	0.251	1.000	Reference	0.262
Afternoon (12:00 ~ 17:59)	1.025	0.960–1.095	0.460	1.031	0.977–1.088	0.270
Night (18:00 ~ 23:59)	0.992	0.927–1.062	0.817	1.001	0.948–1.057	0.961
Dawn (00:00 ~ 05:59)	1.095	0.983–1.128	0.099	1.056	0.981–1.137	0.145

In the bicycle-related injury group, the rural area factor had an adjusted OR of 1.078 (CI 1.021–1.140) compared to the urban area.

## Discussion

This was the first nationwide study to analyse the incidence and characteristics of two-wheeled vehicle-related injuries. This study is more representative than previous studies because it involved all regional emergency medical centres, local emergency medical centres, and local emergency medical rooms in South Korea. With increasing elderly population, South Korea is an ageing society; therefore, comparing such differences between the young and the elderly is very meaningful. When humans age, their brains shrink, including areas related to physical activity [25, 26]. Older people are more susceptible to two-wheeled accident-related injuries because they find it difficult to keep their balance and can fall easily owing to slowed reflexes. Senile degeneration causes serious medical problems and is a major factor that amplifies damage in the elderly by accelerating the decline in cognitive and motor skills due to physiological aging [27]. As life expectancy increases, injuries in the elderly and the resulting disabilities trigger a vicious cycle that causes additional medical problems by reducing the quality of life and physical activity [28].

Accident mortality from automobile accidents has declined in South Korea [29]. The increase in the survival rate has been attributed to the development of automobile technology, the expansion of road infrastructure, the improvement of emergency medical services, [30, 31] and the increase in the healthy elderly population [32]. The number of accidents has not decreased significantly. Indeed, the accident and mortality rates related to bicycles and motorcycles have been steadily increasing [18]. In this study, there were 584 deaths among 65,648 emergency visits due to bicycle-related injuries and 1585 deaths among 87,855 emergency visits due to motorcycle-related injuries over the three-year period.

Bicycles and motorcycles are popular vehicles worldwide. Riding a bicycle strengthens the muscles, increases physical activity, and promotes balance. In addition, bicycles are used as transportation because they are cheaper and easier to use than other means of transportation [33, 34]. The motorcycle is a convenient transportation mode, especially for travelling on roads that are difficult to access by car, and an enjoyable leisure activity for those who appreciate its speed.

Many studies have shown that wearing a helmet significantly reduces brain injury in the event of a two-wheeler accident [35–37]. However, 74.6% of adult riders involved in bicycle accidents and 22.9% of riders involved in motorcycle accidents were not wearing a helmet. Tak reported that motorcycle helmets were used more by the elderly than by younger riders (60.16% vs 51.87%), whereas bicycle helmets was not used as much by the elderly (2.37% vs. 4.82%) [38]. However, in this study, the rate of helmet use in the elderly group for both bicycle and motorcycle riders was lower than that of younger riders (10.5% vs 29.9% for bicycle riders and 62.4% vs 80.7% for motorcycle riders) (Table 2). The analysis of patients visiting the ED revealed that more head and neck injuries occurred in motorcycle accidents than in bicycle accidents, which emphasises the importance of wearing a helmet when riding a motorcycle. In motorcycle accidents, helmet users showed a lower incidence, compared with those who did not wear a helmet, of head injury (mortality: 42% vs 69%) [36] and neck injuries (mortality: 2.4% vs 7.1%; *p* < 0.01) [39].

The elderly group showed fewer injuries during the night-time and dawn periods than younger riders in this study. This indicates that the lifestyles of elderly riders involved fewer outdoor activities at these times, which was consistent with the findings of a previous study that showed fewer traffic crashes caused by elderly riders than by younger riders during the same periods [40].

There was a high percentage of males associated with two-wheeled vehicular damage: 73.6% of bicyclists (71.3% of younger riders, 81.2% of elderly riders) and 89.1% of motorcyclists (90.9% of younger riders, 82.1% of elderly riders). Hsieh et al. reported that female motorcycle riders had different injury characteristics (lower ISS and in-hospital mortality) and presented with bodily injury patterns that differed from those of their male counterpats [41].

The incidence of two-wheeled vehicular accidents per million population was compared in urban and rural areas. There were usually more two-wheeled vehicle-related adult trauma patients per 100,000 population in urban areas than in rural areas; however, there were more motorcycle-related trauma patients per 100,000 population in rural areas in the elderly group (54.8/100,000 population in urban areas and 118.1/100,000 population in rural areas). This could be attributed to the greater suitability of motorcycles for rural roads and consequently wider use as the mode of transportation than cars. It is economically difficult to own a vehicle in the countryside. Many farm roads are narrow and unpaved, hindering vehicular access, and there is a lack of public transportation options in rural areas. Hence, motorcycles are often used in rural areas. The Korean population, especially the rural population, is ageing rapidly, with a large proportion of elderly people (aged≥65 years) in rural areas [42]. In 2019, the elderly accounted for 46.6% of the population involved in farming, 39.2% in fishing, and 44.8% in forestry in rural areas [43]. Hence, policy efforts, such as the improvement of rural roads, the provision of safe and affordable alternative means of transportation, and the reinforcement of safety education for the elderly when using two-wheeled vehicles are necessary.

Among patients who visited the ED because of bicycle and motorcycle accidents, elderly riders mainly injured their head and neck and chest regions, which could be attributed to the lack of helmet use and poor agility. However, helmet use may not always prevent head injuries [44]. Injuries of the pelvis, upper extremities, and lower extremities were common in elderly riders. In bicycle-related accidents, younger riders usually injured their upper extremities, whereas elderly riders had more lower extremity injuries. Both younger and elderly riders fractured their forearm (27.1% vs 15.1%), and fewer younger riders compared with elderly riders injured their lower extremities (3.6% vs 5.9% tibia/fibula injuries and 2.9% vs 11.1% femoral injuries).

Fractures of the lower extremities were more common than those of the upper extremities in bicycle-related accidents, [36] and injuries of the pelvic region and extremities were the most common in elderly riders [45]. Liu et al. reported that cyclists had a higher rate of femoral injuries than motorcyclists [46]. Our study also showed more femoral fractures in bicycle-related accidents (5.0%) than in motorcycle-related accidents (4.2%). This was due to femoral fractures in 11% of elderly riders with bicycle injuries compared to 2.9% in younger riders. However, in this study, in motorcycle accidents, the elderly group showed the most forearm fractures (12.0%) and tibia/fibular fractures (10.0%), which was consistent with the results reported by Chung et al. [45] While younger riders are thought to have more injuries to their upper extremities due to their faster response time and defensive use of their arms when falling from two-wheeled vehicles, the elderly are more likely to be crushed by the vehicle due to their slow response time. Therefore, injury patterns depend both on the mechanisms of the accident and the age of the riders [44]. Because elderly riders employ fewer defensive postures during falls, their injuries are more severe than those of younger riders. In the event of a two-wheeled vehicle-related accident, the elderly have higher rates of ISS ≥ 16, admission, and mortality than younger riders. Elderly motorcycle-related trauma patients had higher ISS, less favourable outcomes, a higher rate of admission to the ICU, and higher mortality than younger adult two-wheeled vehicle riders. As motorcycles are faster and heavier than bicycles, riders suffer more injuries in motorcycle accidents.

Among the reasons for hospital admission through the ED, head and neck injuries were the leading cause (48.8% for younger riders and 65.4% for elderly riders in bicycle accidents; 53.3% for younger riders and 73.3% for elderly riders in motorcycle accidents), while injuries to the extremities accounted for the largest proportion of patients who were hospitalised.

Among hospitalised patients, the most common sites of limb fractures in bicycle injuries were the forearm (10.3%) and femur (8.4%), and the most common sites of limb fractures in motorcycle injuries were the tibia/fibula (11.8%) and forearm (9.0%). Femoral fractures were almost three times more frequent in elderly bicyclists than in younger bicyclists. Elderly inpatients had higher ISS and mortality and longer hospital stays than younger riders involved in two-wheeler accidents. Moreover, elderly patients with motorcycle-related injuries had more severe injuries and longer hospital stays than elderly bicycle-related trauma patients. Therefore, elderly riders suffered worse injuries and showed worse outcomes than younger riders in two-wheeled vehicular accidents. Two-wheeled vehicle-related trauma patients tend to develop fractures of the lower extremities as they age. Meessen et al. showed that the mortality rates of elderly riders after a proximal femoral fracture 1, 6, 12, and 24 months after the accident were 4.7, 16, 20.7, and 30.4%, respectively [47]. Therefore, measures to reduce two-wheeled vehicle-related injuries should be considered from various angles for the growing elderly population. Attention should be paid not only to head and neck injuries, but also to lower extremity injuries in the ED.

From 2016 to 2018, the number of elderly involved in bicycle and motorcycle accidents increased, and the hospitalisation rate, mortality rate, and LOS in the hospital were all higher than those observed for younger riders.

South Korean society is predicted to become a super-aged society, with the elderly accounting for more than 20% of the population by 2026; hence, two-wheeled vehicle-related injuries are expected to increase further among the elderly.

This study can serve as the basis for preparing a plan to reduce the incidence and severity of injuries in the elderly by analysing the characteristics of trauma.

However, this study has several limitations. It did not consider other factors that affect the rates of two-wheeled vehicular accidents, such as weather, pavement, flow of traffic, roadworthiness of the two-wheeled vehicle, and medical costs [31, 32, 44]. Homma et al. suggested that alcohol consumption increased medical cost and independently affected injury severity [2]; however, alcohol consumption was not considered in this study.

This study did not investigate whether the purpose of the two-wheeled vehicles was for leisure, commuting, or work. A total of 4267 (2.0%) patients were excluded because of the poor fidelity of the transferred data. This may have affected the results.

In this study of two-wheeled vehicle related injuries, the analysis of the entire population without access to the risk-exposed group was a limitation. According to data from the National Police Agency, [48] the elderly group held 37.7% of car licenses and 2.3% of motorcycle licenses, whereas the younger group held 77.1% of car licenses and 0.36% of motorcycle licenses. However, because a group with a license could not be viewed only as a risk group, each group had a license and might not operate. There could be many cases of people, not operating or owning a device. It was also because there was a possibility of a passenger injury.

Finally, re-admission or post-outpatient hospitalisation after discharge from the ED was not monitored.

## Conclusion

Two-wheeled vehicle-related injuries in the elderly were associated with higher ISS, hospitalisation rates, length of hospital stay, and mortality than in younger riders. Helmet use was low among the elderly. Injuries to the head and lower extremities, especially femoral fractures, were more common in elderly bicyclists. Analysing the characteristics of injuries in the elderly related to two-wheeled vehicles-related accidents can be the basis for planning to reduce and prevent injuries in elderly riders.

## Data Availability

The datasets used and/or analysed during the current study are available from the corresponding author on reasonable request.
